# Three Phages from a Boreal Lake during Ice Cover Infecting *Xylophilus*, *Caulobacter*, and *Polaromonas* Species

**DOI:** 10.3390/v15020307

**Published:** 2023-01-22

**Authors:** Elina Laanto, Hanna M. Oksanen

**Affiliations:** 1 Molecular and Integrative Biosciences Research Programme, Faculty of Biological and Environmental Sciences, University of Helsinki, 00014 Helsinki, Finland; 2 Department of Biological and Environmental Science, Nanoscience Center, University of Jyväskylä, 40014 Jyväskylä, Finland

**Keywords:** virus isolation, phage genomics, freshwater phage, *Xylophilus* phage, *Caudobacter* phage, *Polaromonas* phage

## Abstract

Although the important role of microbes in freshwater is well understood, studies on phage–host systems in such environments during ice cover are completely lacking. Here, we describe the isolation and characterization of three new bacteriophages infecting *Xylophilus* sp., *Caudobacter* sp., and *Polaromonas* sp. from freshwater samples taken under the ice cover of Lake Konnevesi, Finland. Lumi, Kuura, and Tiera bacteriophages have tailed icosahedral virions and double-stranded DNA. Lumi is a siphophage with a genome of 80,496 bp, and Kuura and Tiera are podophages, and their genomes are 43,205 and 45,327 bp in length, resembling viruses in the class *Caudoviricetes*. Their host ranges were very limited among the winter-isolated bacterial strains from Konnevesi, each infecting only their own hosts. They can infect efficiently at 4 °C, showing that they are adapted to living in lake water under ice cover. Analysis of the viral genome sequences showed that a significant number of the gene products of each virus are unique, indicating that there is unexplored viral diversity in freshwaters. To our knowledge, Lumi and Tiera are the first phages isolated on the *Xylophilus* sp. and *Polaromonas* sp. strains, allowing their exploitation in further studies of freshwater bacterial–phage interactions.

## 1. Introduction

Bacterial viruses, phages, are numerically the largest group of biological entities in the biosphere. Especially, aquatic viruses have been central model systems for the past few decades in studying phages and their ecology. As 97% of Earth’s water is saline ocean water, studies of aquatic phages and their dynamics have been more concentrated on marine systems. Indeed, the importance of marine viruses for aquatic biochemical cycling has been well-demonstrated, e.g., [1,2]. The biochemical cycling is not affected only through viral lysis, but prokaryotic viruses can also acquire and express auxiliary metabolic genes (AMGs). In addition, it has been recognized that viruses have a significant role in freshwater carbon cycling [3]. Freshwater systems are highly sensitive to environmental changes. Many freshwater environments have seasonal variations, and some water bodies remain ice-covered for most of the winter. This is typical for freshwater environments in the northern hemisphere. Under ice, freshwater microbial communities in winter are actively growing, not being dormant as might be expected, and their microbial diversity is even higher than during ice-free time periods [4,5]. The abundant microbes serve as hosts for viruses to multiply under ice. In numbers, a milliliter of freshwater typically contains 10^5^–10^7^ prokaryotes and 10^6^–10^8^ viruses [6]. Microbial mortality caused by viruses diverts the organic matter from animal trophic levels. It is likely that freshwater phages can be a source for a deeper understanding of the phage world and the role of phages in these ecosystems. For example, a completely new phage type was recently isolated from a boreal lake with an abundant freshwater bacterium host, *Flavobacterium* [7]. In addition, metagenomic studies have shown, e.g., the abundance of antibiotic resistance genes in freshwater phage genomes [8], the domination of unknown ssDNA viruses in Arctic freshwater viromes [9], and the potential ability of large freshwater phages to augment the oxidation of methane [10]. However, the experimental virus–host system-based data can provide invaluable insights for interpreting sequence data providing details such as gene function. 

Overall, knowledge of boreal lake microbial dynamics, especially during wintertime is scarce, and there are no data on the phages during this period. Further, the phages active during the cold period can carry genes encoding for cold-active enzymes and thus be potentially beneficial for biotechnological applications. Here, we take a step forward in understanding the phage–host systems during winter and under ice cover by isolating three phages and their hosts from a boreal lake in Central Finland. The sampling was conducted during the ice cover. We characterized the host range of the three phages among the isolated bacteria and show that phage genomes are unique, reflecting the undiscovered abundance of viruses in freshwater environments.

## 2. Materials and Methods

### 2.1. Isolation and 16S rRNA Gene Sequencing of Bacteria 

Bacteria were isolated from a water sample collected on 26 December 2006 from Lake Konnevesi in Central Finland (N 62°32′29.663″ E 263°5′47.813″) by spreading 100 µL of water on a one-fifth diluted LB medium [11] (1/5 LB) agar plate (1%, *w*/*v* agar) and grown for two days. All cultures were performed in 1/5 LB broth prepared in tap water at room temperature (RT) unless otherwise indicated. Single colonies were purified by forming consecutive pure cultures until a single colony culture was obtained. For bacterial identification, the 16S rRNA gene was partly amplified and sequenced. A colony from a pure culture was suspended into 50 µL of dH_2_O, and 1 µL of suspension was used as a template for PCR. Primers fD1 and rD1 [12] and Biotools DNA polymerase (Biotools B&M Labs S.A., Madrid, Spain) were used for amplification. For strains B20, B21, and B54, a primer pair of 27f (5′-AGAGTTTGATCMTGGCTCAG-3′) and 518r (5′- ATTACCGCGGCTGCTGG -3′) was used. Purified PCR products (PCR purification kit, Qiagen, Hilden, Germany) were sequenced using ABI Prism 3130xl (Applied Biosystems, Waltham, MA, USA) and BigDye3.1 (Thermo Fisher Scientific, Waltham, MA, USA). Sequences were searched against the EzBioCloud database [13] during the autumn of 2021. Alignment was created using Clustal Omega in Geneious software Prime version 2022.2 (Biomatters Ltd., Auckland, New Zealand), and a tree was built with the Neighbor-joining method and the Jukes–Cantor model.

### 2.2. Phage Sampling and Enrichment

First, phage isolation was tried using samples that were collected on 1 May, 24 September, and 8 October 2007 by a direct plating method using 300 µL water samples with 1–2-day-grown bacteria. Plating was performed using the double agar layer method using 1/5 LB top agar (0.5%, *w*/*v* agar). The phages were isolated from a sample collected on 17 February 2008 with an enrichment method: LB medium was mixed with the filtered (0.45 µm, Nalgene) water sample in a 1 to 5 ratio, and 1–2-day-grown bacterium culture was added (1:10) and grown until turbid. Enrichment culture (300 µL) was plated using the double agar layer method. Plaques were picked after two days of incubation, and three rounds of plaque purification were performed for each isolate. Bacteria and phages were stored in the cold room or at –80 °C with 20% (*v*/*v*) glycerol for a longer time.

### 2.3. Phage Culturing and Host Range Analysis

High titer lysates were prepared from plates with semi-confluent or confluent lysis by adding 4 mL of medium per collected top agar and incubated with shaking for 2 h at RT (~22 °C). The lysate was centrifuged to remove agar and bacteria, filtered (0.22 µm), and stored in a cold room. The plaque formation of viruses in the cold room was analyzed by growing titration plates at +4 °C for 2–3 weeks (*n* = 3). Titration plates grown at RT were used as controls (*n* = 3).

For host screening, undiluted and diluted (10^−1^–10^−7^) phage lysates were placed in two parallel drops of 10 µL on two plates. The soft agar with added bacterial culture was solidified for half an hour at room temperature before the drops were placed. The growth medium and original phage infecting the host were used as controls. 

### 2.4. Phage Purification and Transmission Electron Microscopy 

For virus purification, the particles were precipitated using 10% (*w*/*v*) polyethylene glycol 6000 and 0.5 M NaCl, which were dissolved for 30 min at 4 °C, and the precipitate was collected by centrifugation (Sorvall rotor F14, 8000 rpm, 30 min, 4 °C). The pellet was suspended in 20 mM potassium phosphate at pH 7.2, and the aggregates were removed by centrifugation (tabletop centrifuge, 7000 rpm, 10 min, 4 °C). The viruses were purified on a 5–20% (*w*/*v*) sucrose gradient in 20 mM potassium phosphate, pH 7.2. (Lumi phage: Sorvall rotor AH629, 24,000 rpm, 30 min, 15 °C; Kuura and Tiera phages: Sorvall rotor TH641, 24,000 rpm, 45 min, 15 °C). The viruses were collected from the sucrose gradients, and their infectivities were determined. For transmission electron microscopy (TEM), phages in a sucrose band were stained on glow-discharged copper grids with 2% phosphotungstic acid for 2 min. Imaging was performed with a JEOL JEM-1400HC microscope at 80 kV (University of Jyväskylä, Jyväskylä, Finland). 

### 2.5. Phage Genome Sequencing and Analysis

The phage genome was extracted from the filtered phage lysate using a method by Santos [14] with modifications. First, 2 mL of lysate was incubated with 4 µg/mL of DNase I (Sigma) and 40 µg/mL of Rnase A (Macherey-Nagel, Düren, Germany) for 30 min at 37 °C, and the phage was precipitated using 0.04 M of ZnCl_2_ for 5 min at 37 °C followed by centrifugation (1 min, 10,000 rpm). The pellet was suspended in 1 mL of TES buffer (0.1 M Tris-HCl pH 8.0, 0.1 M EDTA, 0.3%, *w*/*v* SDS), and 0.8 mg/mL of Proteinase K (Roche) was used for the dissociation of the phage particles. The DNA was bound to a column of a commercial genomic DNA extraction kit (GenElute Bacterial Genomic DNA kit, Sigma, St. Louis, MO, USA; GeneJET Genomic DNA purification Kit, Thermo Fisher Scientific, Waltham, MA, USA) with 2 M GuHCl and 20% (*v*/*v*) EtOH and washed and eluted according to the manufacturer’s instructions. The sizes of the genomes were analyzed by agarose gel electrophoresis using purified uncut genomes, the Lumi genome cut with XbaI, and the Kuura and Tiera genomes cut with BamHI.

The genome sequencing was performed commercially at Novogene with Illumina NovaSeq and paired-end 150 bp sequencing. The resulting reads were trimmed with BBDuk and assembled with Velvet [15] in the Geneious platform (Biomatters Ltd.). To determine the genome ends, trimmed reads were run in PhageTerm [16]. The rearranged genome was annotated first using RAST [17]. In addition, GenemarkS was employed [18]. Blastp [19] and HMMER [20] searches were performed for predicting the function, and additional HHPred [21] searches were performed for some of the predicted ORFs. Genome alignments were created with EasyFig [22] employing Blastx. 

## 3. Results

### 3.1. Bacterial Species Isolated under Ice Cover 

**Figure 1:**
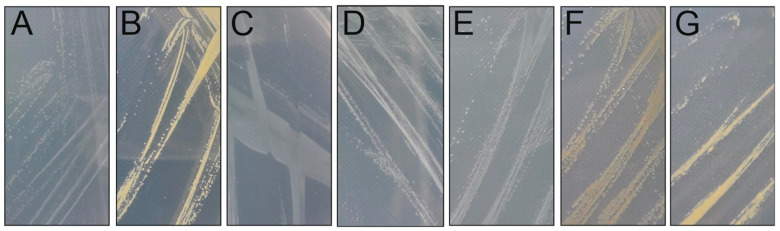
Colonies of (**A**) *Xylophilus* sp. B14, (**B**) *Caulobacter* sp. B15, (**C**) *Polaromonas* sp. B16, (**D**) *Pseudomonas* sp. B20, (**E**) *Herbaspirillum* sp. B21, (**F**) *Flavobacterium* sp. B28, (**G**) *Sphingomonas* sp. B54 grown at RT on 1/5 LB plates.

Using 1/5 LB medium and room temperature, seven bacterial strains were isolated from a lake water sample taken during ice cover (Table 1). They all formed colonies in 2–3 days on a plate (Figure 1).

**Figure 2:**
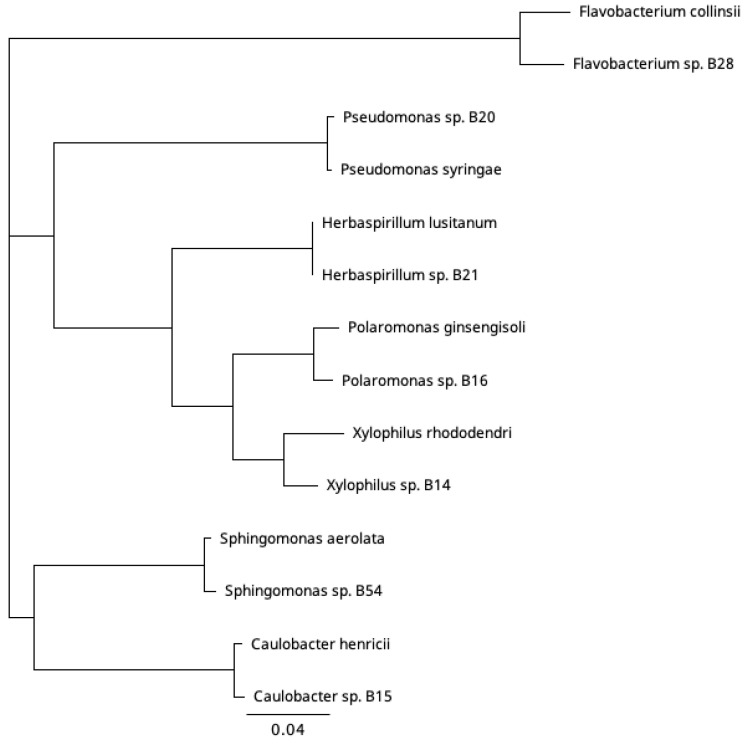
Phylogenetic relationship based on partial 16S rRNA sequence among the studied bacteria isolated from a freshwater sample collected during ice cover. Best similarity hits to bacterial strains with species names were included in the analysis (species strain:accession: *Caulobacter henricii* ATCC 15253: AJ227758, *Flavobacterium collinsii* 983-08: HE612088, *Herbaspirillum_lusitanum*_P6-12: AJHH01000137, *Polaromonas ginsengisoli* Gsoil 115: AB245355, *Pseudomonas syringae* KCTC 12500: KI657453, *Sphingomonas aerolata* NW12: AJ429240, *Xylophilus rhododendri* CJ1-R5: MN912104 Alignment Clustal Omega, Neighbor-joining method in Geneious Prime 2022.2. (Biomatters Ltd.).

A long partial 16S rRNA was obtained for the three bacteria, for which a phage was isolated, and a shorter sequence was obtained for the rest (Table 1). We classified the bacterial isolates at the genus level, using a threshold of 95% 16S rRNA gene sequence identity to representative species with a complete 16S rRNA gene sequence available in the GenBank database. The isolates were unique among themselves and belonged to seven genera: *Xylophilus*, *Caulobacter*, *Polaromonas*, *Pseudomonas*, and *Herbaspirillum* in the phylum Proteobacteria, *Flavobacterium* in the phylum Bacteroidota, and *Sphingomonas* in the phylum Pseudomonadota (Figure 2). The closest hit of the bacterial isolate B14 was *Xylophilus rhododendri* (98.6% pairwise identity) [24]. Gram-negative *Xylophilus* species are typically plant pathogens. For isolate B15, the closest hit was to *Caulobacter henricii* (99.7% pairwise identity) [25]. *Caulobacter* are stalked bacteria associated with submerged surfaces and are abundant in freshwater. For isolate B16, the closest hit was to *Polaromonas ginsengisoli* (98.9% pairwise identity) originating from soil [26]. In addition to soil, Gram-negative *Polaromonas* bacteria have been found, e.g., from marine water and sediment, and some of them are psychrophiles [27,28]. Other bacteria isolated from the same sample and used in this study were identified by shorter 16S rRNA sequences: *Pseudomonas* sp. B20 (*Pseudomonas syringae* strain KCTC 12500, 99.4% pairwise identity), *Herbaspirillum* sp. B21 (*Herbaspirillum lusitanum*, 100% pairwise identity), and *Sphingomonas* sp. B54 (*Sphingomonas* LMKH_s Leaf208, 99.5% pairwise identity). Another bacterium that originated from the same sample but has been previously published was *Flavobacterium* sp. B28 [23].

### 3.2. Three Unique Tailed Double-Stranded DNA Phages Were Isolated from a Boreal Lake Water Sample during Ice Cover

**Figure 3:**
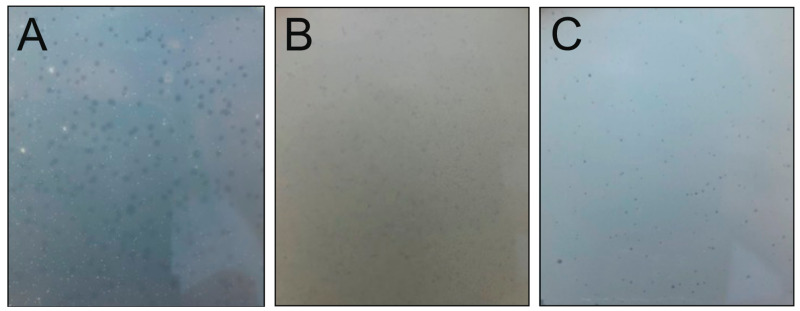
Plaque morphologies of (**A**) Lumi phage, (**B**) Kuura phage, and (**C**) Tiera phage grown on 1/5 LB at room temperature.

Using samples collected from Konnevesi during the ice-free period (May, September, and October 2007 samples), we were unable to detect any plaque on the bacterial strains (Table 1). When samples were taken in February 2008 under ice cover and cultured with bacterial isolates isolated from the same sampling site, three phage isolates, Lumi, Kuura, and Tiera, were obtained on *Xylophilus* sp. B14, *Caulobacter* sp. B15, and *Polaromonas* sp. B16 (Table 2). The viruses formed plaques after 1–2 days of incubation at RT and the plaques were clear or slightly turbid and 0.5–2 mm in diameter (Figure 3). The phages grown by the double-layer agar method produced high titer agar stocks of 10^8^–10^10^ pfu/mL (Table 2). The plaques were sometimes hazier, which made it difficult to accurately count the number of plaques. The phages were also able to form plaques at cold temperatures. The plaque formation took 15–20 days at 4 °C, with a similar level or slightly lower number of plaques than at RT (Table 2). The pfu/mL at 4 °C was on average 2.7 × 10^9^ for Lumi, 2.3 × 10^10^ for Kuura, and 1.0 × 10^10^ for Tiera (plaque numbers from Tiera are an estimation). The appearance and size of the Kuura plaques were similar between cold and RT cultivation, whereas Lumi and Tiera formed turbid and less clear plaques at 4 °C. All three bacterial host strains of the phages formed a proper culture in the plaque test during cold culturing. In further work, room temperature was used. By cross-testing the seven winter-sampled bacterial species (Table 1) with the three phages, we revealed that Lumi, Kuura, and Tiera could form plaques only on their original host strains. 

**Figure 4:**
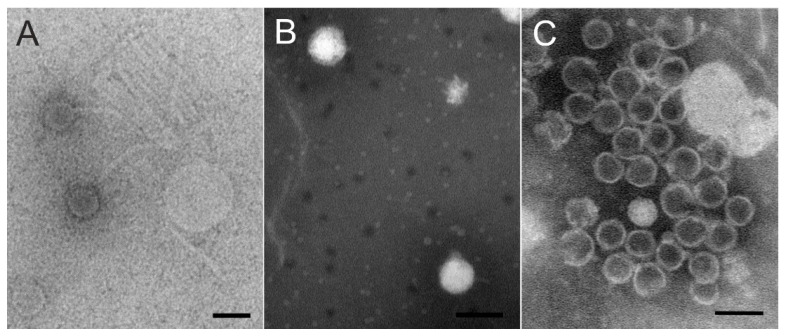
Negatively stained phage particles under transmission electron microscope. (**A**) Lumi (**B**) Kuura and (**C**) Tiera. Scale bar is 100 nm.

Viruses were purified by polyethylene glycol–NaCl precipitation and rate zonal ultracentrifugation in sucrose. In the purification of phages Kuura and Tiera, two light-scattering zones were formed, the lower one with more infectivity (~10^8^–10^9^ pfu/mL). The purification of Lumi resulted in a diffuse light scattering band with a titer of ~10^6^ pfu/mL. Purified phages were used for negative staining and TEM. Particles of the Xylophilus phage Lumi had a siphovirus-like capsid and tail structure under TEM (Figure 4A; see also below). The tails were long and flexible and mostly unattached from the capsids. The morphologies of the Caulobacter phage Kuura and Polaromonas phage Tiera particles were podovirus-type with a short tail (Figure 4B,C). In the TEM images, most of the Tiera particles had lost their DNA and only a few DNA-containing particles were visible. The capsid diameter was estimated to be approximately 65 to 70 nm.

#### 3.2.1. Phage Lumi Infecting *Xylophilus* sp. B14 Shows Relatedness with Achromobacter Phage JWF 

**Figure 5:**
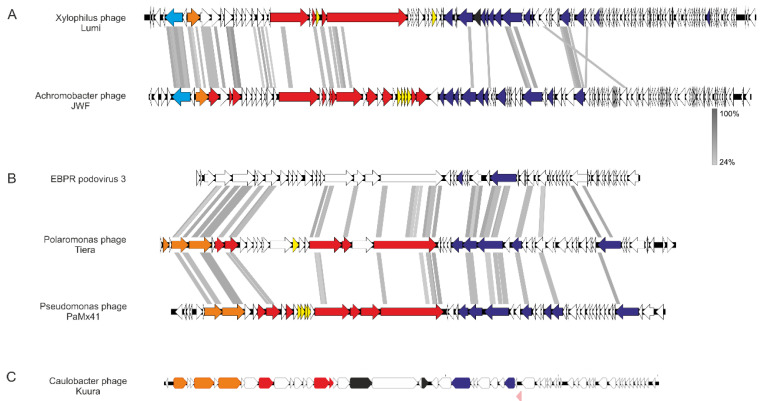
Genome organizations of (**A**) Lumi, (**B**) Tiera, and (**C**) Kuura phages and their related phages. In (**A**), Xylophilus phage Lumi (80,496 bp in length) and Achromobacter phage JWF (81,541 bp). Both have 118 predicted open reading frames (ORFs). In (**B**), the genomes of Polaromonas phage Tiera (45,327 bp) in the middle and related EBPR podovirus 3 on top (a partial sequence, 38,991 bp, assembled from metagenome) and Pseudomonas phage PaMx41 (43,490 bp) in the bottom. In (**C**), the genomic organization of Caulobacter phage Kuura (43,813 bp), which is unique. Putative functional genes are color-coded: Light blue corresponds to primpol, orange packaging, red structural, yellow genes related to host lysis, black transglycosylases, blue replication, and pink repressor. White denotes hypothetical protein. Functions defined in other phages [32,33,34] were used as references. Alignments were created with Easyfig [22] employing Blastx. Grey areas between the genomes indicate detected amino acid identity; the level is indicated with a gradient scale in between (**A**) and (**B**).

The siphovirus-like morphology of the Xylophilus phage Lumi was confirmed also from the genome. Sequencing resulted in an 80,496 bp-long genome with a GC content of 57% and 118 predicted ORFs. The XbaI-cut genome was digested according to the sequence to confirm the genome size. PhageTerm analysis for the genome ends detected long direct terminal repeats (2298 bp). All three phage genomes were dsDNA (Figure 5), which were confirmed by cutting with restriction endonucleases, and included ORFs encoding small and large terminase subunits, which are hallmark genes for tailed dsDNA bacteriophages that package their genomes into a preformed procapsid [29,30]. Although Lumi is a unique phage, ORF searches with Blastp suggested that Lumi shares some similar ORFs with the Achromobacter phage JWF, a siphovirus originating from sewage [31,32]. Especially putative packaging, structural, and replication genes showed similarities between the two phages. Significant hits to different genes in JWF were for example to large terminase, received with HMMER and Blastp (97% coverage, E-value 2 × 10^149^ in Blastp), and DNA polymerase (98% coverage, E-value 0 in Blastp). Indeed, the genome size and number of ORFs in Lumi are similar to JWF (81,541 bp with 118 ORFs). The host species of both phages, *Xylophilus* and *Achromobater*, belong to the same order Burkholderiales. In addition, the Blastp search received several hits also to other bacterial species belonging to the same order (Supplementary Table S1). Putative lysis-related genes were identified between the genes encoding predicted structural and replication-related functions, but those were non-homologous to those of JWF. 

#### 3.2.2. Two Podoviruses Were Isolated, Phage Kuura Infecting *Caulobacter* sp. B15 and Phage Tiera Infecting *Polaromonas* sp. B16

Two of the phages, Caulobacter phage Kuura and Polaromonas phage Tiera, displayed a podovirus-like morphology under TEM (Figure 4B). The sequencing of the phage Kuura genome resulted in a 43,205 bp-long sequence with a %GC of 63 and 61 predicted ORFs (Figure 5C). Cutting the Kuura genome with BamHI resulted in correctly sized fragments verifying the assembly of the genome. Genome end analysis with PhageTerm detected direct terminal repeats (606 bp). Blastp-predicted ORFs of Kuura received hits only to a few phages (16 ORFs, out of which, 13 were metagenomes) in addition to bacterial proteins (Supplementary Table S1). Additionally, only three ORFs received a hit to the host bacterium Caulobacter. Over half of the predicted ORFs (37 out of 61) did not receive any significant similarities in the Blastp search. A large terminase, lytic transglycosylase, and a DNA polymerase were identified with Blastp, and additional searches with HHPred identified also a small terminase, portal protein, structural proteins, and a putative repressor (Figure 5C, Supplementary Table S1). Indeed, the plaques of Kuura were also turbid. Although there were no related phages detected for Kuura, the predicted ORFs of phage Tiera received some hits to other podoviruses (Figure 5B, Supplementary Table S1). Especially, MMER searches revealed several hits to the structural and packaging proteins of *Pseudomonas* aeruginosa-infecting podophages isolated in Mexico (e.g., PaMx41) and an EBPR Podovirus, a partial podovirus genome assembled from a metagenome [33,34]. The Tiera genome was 45,327 bp long with a %GC of 48 and included 55 predicted ORFs. Approximately half of the predicted ORFs (23/55) in the Tiera genome did not receive any hits in the Blastp search. Putative small and large terminase and portal proteins were identified from the left side of the genome. These were followed by two putative capsid proteins (major capsid proteins) (Figure 5B).

## 4. Discussion

Freshwater environments are subjected to dramatic seasonal changes. Although the ice cover time, especially in the northern hemisphere, can be significantly long, little is known about the phage–host dynamics during this period. However, the microbial community goes through dramatic shifts in abundance and diversity [4]. This indicates that the viral community is also highly diverse and active during the cold winter months and can play an important role in shaping microbial communities. Here, we investigated the cultivable phage–host systems under the ice cover in a boreal lake. Out of the seven isolated bacteria, three species (*Flavobacterium*, *Sphingomonas*, and *Polaromonas)* belonged to lineages that have been previously described as common glacier-ice lineages [35]. For three bacteria, *Xylophilus* sp., *Caulobacter* sp., and *Polaromonas* sp., a phage isolate was obtained. All isolated bacteria belong to the phyla Proteobacteria and Bacteroidetes and are among the dominant phyla in metagenomes from boreal freshwater lakes with clear water [36]. To our knowledge, both *Xylophilus* sp. and *Polaromonas* sp. phages are the first descriptions of phages infecting these species. 

The Xylophilus phage Lumi, the Caulobacter phage Kuura, and the Polaromonas phage Tiera were all able to form plaques at 4 °C and at room temperature without any significant difference in PFU/mL between the two temperatures. This shows that the phage–host systems are active also in conditions relevant to those under ice cover. Out of the seven bacterial isolates derived from the same site, phages were able to infect only their own hosts. This would be suspected as all the bacterial isolates belong to different genera. All three phages were morphologically detected to have a head–tail structure. Lumi showed a siphovirus-like long and flexible tail, and Kuura and Tiera showed podovirus-like morphology with a short tail.

In addition to the morphological evidence, the genomes also showed a dsDNA nature. The Xylophilus phage Lumi genome showed a similar size, organization, and number of predicted ORFs to the Achromobacter phage JWF originating from sewage [31]. Searches of the ORFs against the database received significant hits to JWF throughout the genome. Indeed, both *Xylophilus* sp. and *Achromobacter* sp. belong to the order Burkholderiales. Out of the three phages, Caulobacter phage Kuura was the most novel in the sense that its genome received very few hits to known phages. Overall, Kuura contained most ORFs that did not receive any hits. Polaromonas phage Tiera received several hits to a partial podovirus genome assembled from metagenomic data from an enhanced biological phosphorous removal reactor (EBPR) [33]. Tiera also showed relatedness with podoviruses, especially *Pseudomonas aeruginosa* podoviruses isolated in Central Mexico from environmental and sewage samples [37]. Additionally, *Polaromonas* and *Pseudomonas* are both members of the phylum, Proteobacteria. 

In addition to the abundance and diversity of viral fractions in freshwaters, the life strategies of phages can be of high relevance. The two major life strategies, lytic and lysogenic, influence, e.g., phage–host dynamics, biogeochemical cycles, gene transfer, and regulate microbial processes, e.g., [38,39]. In addition, phages can have alternative life cycle strategies, e.g., pseudolysogeny or chronic infection [40]. We did not investigate in detail the life cycle preferences of the phages, but phage Kuura showed turbid plaques, and a putative repressor was detected from its genome, both of which could imply the temperate nature of the phage. The others, Lumi and Tiera had clear plaques, and their genomes showed no sign of the genes of the temperate phages. However, the relatedness of Lumi with the temperate Achromobacter phage JWF may indicate that Lumi could also be a prophage. JWF genes have several hits to unannotated putative prophage regions in Burkholderia species [32]. Similarly, Blastp searches received several hits between Lumi and Burkholderia (in parallel to JWF), especially around the putative structural and replication genes. With three phage isolates, it is impossible to make any generalizations about the frequency of either life cycle during ice cover, but it is possible that both lytic and lysogenic phages are present during this period.

Our study shows that the isolation of phages from a boreal lake during ice cover is possible. Moreover, the characterized phages can improve the analysis of metagenomic data by introducing biological connections to the undescribed viral genetic data. However, in order to make any conclusions on what type and what time certain phage–host systems are active under ice cover, more broad (including different freshwater sources) and periodical sampling and data are needed. To conclude, this research is a step further in the understanding of freshwater microbes and their viruses, especially during ice cover, which has been understudied.

## Figures and Tables

**Table 1:** Properties of the bacterial strains used in the study.

Strain	Colony Properties	16S rRNA Partial Sequence Accession ID and Length (bp)	Reference
*Xylophilus* sp. B14	Pale, opalescent	OP984743 (1429)	This study
*Caulobacter* sp. B15	Matte, yellow	OP984744 (1381)	This study
*Polaromonas* sp. B16	Greenish, pale, matte	OP984745(1424)	This study
*Pseudomonas* sp. B20	Greenish, pale, matte	OP984746 (478)	This study
*Herbaspirillum* sp. B21	Pale, opalescent	OP984747 (464)	This study
*Flavobacterium* sp. B28	Transparent, yellow	FR696328 (853)	[23]
*Sphingomonas* sp. B54	Matte, yellow	OP984748 (363)	This study

**Table 2:** Freshwater lake viruses isolated from under ice cover.

Phage ^1^	Lumi	Kuura	Tiera
**Host bacteria**	*Xylophilus* sp. B14	*Caulobacter* sp. B15	*Polaromonas* sp. B16
**Plaque diameter (mm) ^2^**	1–2	0.5	0.5–1
**Titer of virus stock at RT (pfu/mL) ^3^**	5.4 × 10^9^ ± 5.0 × 10^9^	2.7 × 10^10^ ± 1.5 × 10^10^	5.0 × 10^9^ ± 1.0 × 10^9^
**Virion morphology**	siphovirus	podovirus	podovirus
**Genome length (bp)**	80,496	43,205	45,327
**%GC**	57	63	48
**Genome sequence accession number**	OQ067477	OQ067476	OQ067478
**Reference**	This study	This study	This study

^1^ Etymology: Lumi is Finnish and means snow, Kuura is Finnish and means frost; Tiera is Finnish and means icy snow accumulation on the bottom of a hoof or shoe. ^2^ Plaques were grown at RT for two days. ^3^ Pfu numbers as mean from three technical repetitions with standard deviation.

## Data Availability

All sequence data produced during the research have been deposited in the GenBank under accession numbers OP984743-OP984748 for the 16SrRNA and OQ067476- OQ067478 for the phage genomes.

## Supplementary Materials

The following supporting information can be downloaded at: https://www.mdpi.com/article/10.3390/v15020307/s1, Supplementary Table S1. Genome analysis from the three phages with Blastp and additional HMMER and HHPred hits to the predicted ORFs.
